# Routine admission urinalysis has low clinical utility in psychiatric hospitalizations

**DOI:** 10.1017/ash.2025.10291

**Published:** 2026-01-26

**Authors:** Tri Pham, Devanshi Patel, Ana Berce, Alice Bewley, Sena Sayood

**Affiliations:** 1 Department of Medicine, Washington University in St. Louis School of Medicine, St. Louis, MO, USA; 2 Washington University in St. Louis School of Medicine, St. Louis, MO, USA; 3 Division of Infectious Diseases, Washington University in St. Louis School of Medicine, St. Louis, MO, USA

## Abstract

We examined the diagnostic utility of urinalyses (UAs) in psychiatric admissions. Admission UAs led to diagnosis of clinical urinary tract infections in 1.7% of cases. Among those treated with antibiotics, inappropriate prescriptions occurred in 71.3% of cases, with increased odds in older age, female sex, positive cultures, and certain psychiatric diagnoses.

## Introduction

Inappropriate antibiotic use (IAU) increases morbidity, healthcare expenses, and antibiotic resistance.^1^ One driver of IAU is treatment of asymptomatic bacteriuria (ASB), and as such, the Infectious Diseases Society of America (IDSA) guidelines recommend against screening for and treating ASB.

Unfortunately, these practices remain prevalent in inpatient psychiatric floors^2,3^ despite long-standing evidence discouraging them.^4^ In these settings, urinalyses (UAs) are typically ordered for evaluation of altered mental status (AMS). However, there is limited evidence supporting a causal link between bacteriuria without localizing symptoms for urinary tract infections (UTIs) and changes in mentation.^5,6^ Further, IDSA guidelines discourage treating ASB, even in adults with AMS, as it has not been shown to confer any clinical benefits.^1^ Given this disconnect between guidelines and current practice, our study aimed to evaluate the utility of admission UAs in psychiatric hospitalizations and characterize its role in contributing to IAU.

## Methods

This retrospective cohort study was conducted at Barnes-Jewish Hospital (BJH) in St. Louis, Missouri; approval was obtained from the Washington University Institutional Review Board prior to study onset. We identified all admissions to BJH-affiliated psychiatry units between January 1, 2020, and December 31, 2024. The following data were abstracted from the electronic medical records (EMR): demographics, comorbidities, symptoms, vital signs, UA results, antibiotic use, and *Clostridioides difficile* (C. diff) test results within 30 days of psychiatric admission. Only the first recorded vital signs following the psychiatric admission were included in the analyses.

We categorized a UA as positive if pyuria (white blood cells >10 per high-power field) was present or if the resulting urine culture (UC) yielded >100,000 colony-forming units of a single organism. Consistent with the IDSA diagnostic criteria, we categorized patients as having a UTI if they had a positive UA and at least one of the following symptoms: dysuria, frequency, urgency, suprapubic pain, costovertebral angle tenderness, or hematuria. We excluded UTIs in patients who presented with AMS (who may be unable to report symptoms) if there were no systemic signs of infection, which included fever to ≥ 38°C, leukocytosis > 12,000 cells/μL, systolic hypotension < 90 mmHg, or ≥ 2 systemic inflammatory response syndrome criteria. We defined admission UAs as those collected within the 48-hour window before and after arrival to the psychiatric floor to account for delays in transfer or urine specimen collection, and because a UA is typically a mandatory criterion prior to admission. Only antibiotics initiated for urinary indications were considered. We considered an antibiotic initiation inappropriate if the patient did *not* meet the diagnostic criteria for a UTI or was *not* pregnant with a positive UA.

The primary outcomes were the proportion of inappropriate antibiotic prescriptions in patients with positive UAs and the proportion of admission UAs leading to diagnosis of a clinical UTI. The secondary outcomes focused on factors associated with IAU among patients with a positive UA, as well as the incidence of C. diff infection (CDI) among asymptomatic patients with a positive UA who received antibiotics inappropriately versus those who did not. Logistic regression models estimated using generalized estimating equations were fitted to identify factors associated with IAU among those without clinical UTIs, accounting for within-patient correlation across multiple encounters. Variables significant at the 10% level were included in the multivariable model. Forward selection and backward deletion were used to determine the best-fit model using a *P* value of .05 as the inclusion/exclusion criterion. Subgroup analysis was conducted with among encounters that had complete UA testing (ie, reflex to microscopy *and* culture rather than microscopy alone). All analyses were performed using R version 4.4.2 (R Project for Statistical Computing, Vienna, Austria).

## Results

**Figure 1.**
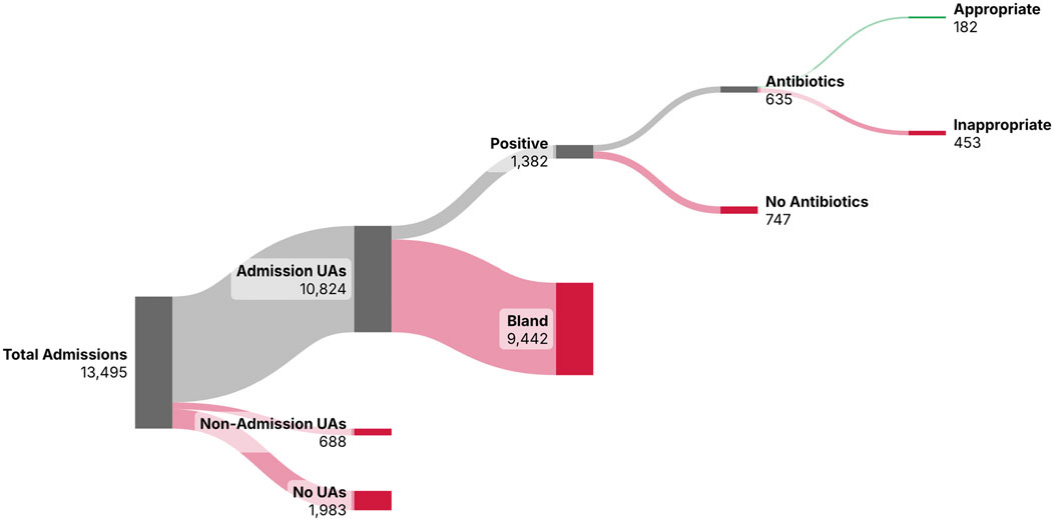
Sankey diagram illustrating the diagnostic utility of admission urinalyses in psychiatric hospitalizations. This Sankey diagram depicts the relative clinical utility of admission UAs for psychiatric hospitalizations. We defined admission UAs as those collected within the 48-hour window before and after arrival to the psychiatric floor to account for delays in transfer or urine specimen collection. We only considered antibiotics initiated for urinary indications. Abbreviations used: UA, urinalysis.

Among 8,891 patients totaling 13,495 psychiatric hospitalizations, 10,824 (80.2%) received admission UAs, with 1,382 (12.7%) having positive UAs. Median age was similar for those receiving UAs (36 years) and those with pyuria (37 years). Of encounters with pyuria, 814 had UC data. Antibiotics were prescribed in 635 (45.9%) encounters with pyuria; among those with positive UAs but no UC data, 402 received antibiotics. Most antibiotic prescriptions were inappropriate (453, 71.3%), including 135 cases of AMS without systemic signs of infection. Only 182 admission UAs (1.7%) led to appropriate treatment (Figure 1), attributable to localizing symptoms or pregnancy (n = 155) or signs of inflammation in those with altered mentation (n = 27).

In our multivariable logistic regression model, factors associated with IAU in patients with positive UAs included older ages >45 years (reference: 18–24 yr), female sex (adjusted odds ratio [aOR] 1.40, 95% CI: 1.07–1.82), and psychiatric diagnoses of schizophrenia/psychotic (aOR 1.69, 95% CI: 1.23–2.31), bipolar (aOR 1.94, 95% CI: 1.23–3.07), and substance use disorders (aOR 1.83, 95% CI: 1.22–2.74), compared to depressive disorders (Table 1). In our subgroup analysis (n = 692) involving encounters with reflex-to-culture testing (excluding those with appropriate antibiotic initiation), a positive UC was significantly associated with IAU (aOR 6.05, 95% CI: 3.09–10.06, robust standard error: 0.26). There were no documented cases of CDI within 30-days after psychiatric admission in either group of interest in our cohort.

**Table 1.** Demographic data and logistic regression model results of factors associated with antibiotic use in patients with positive urinalyses without urinary indication for antibiotics

Variables	Antibiotics received inappropriately (n = 453)	No antibiotics received (n = 747)	uOR (95% CI)	*P*-value	Robust SE	aOR (95% CI)	*P*-value	Robust SE
Age group, n (%)								
18–24	50 (27.17)	134 (72.83)	REF			REF		
25–34	117 (34.62)	221 (65.38)	1.38 (0.93–2.06)	0.11	0.20	1.27 (0.85–1.91)	0.24	0.21
35–44	83 (34.44)	158 (65.56)	1.39 (0.91–2.11)	0.13	0.21	1.26 (0.81–1.94)	0.30	0.22
45–54	85 (44.74)	105 (55.26)	2.19 (1.42–3.38)	**<0.01**	0.22	2.05 (1.31–3.21)	**<0.01**	0.23
55–64	69 (43.40)	90 (56.60)	2.02 (1.28–3.17)	**<0.01**	0.23	1.69 (1.06–2.68)	**0.03**	0.24
65+	49 (55.68)	39 (44.32)	3.37 (1.98–5.73)	**<0.01**	0.27	2.76 (1.61–4.75)	**<0.01**	0.28
Sex assigned at birth, n (%)								
Male	130 (33.25)	261 (66.75)	REF			REF		
Female	323 (39.93)	486 (60.07)	1.31 (1.02–1.69)	**0.04**	0.13	1.40 (1.07–1.82)	**0.01**	0.14
Race/ethnicity, n (%)								
Non-Hispanic White	187 (40.56)	274 (59.44)	REF			--	--	
Black	234 (35.4)	427 (64.6)	0.80 (0.62–1.02)	0.07	0.13	--	--	
Asian and other Pacific islander	7 (53.85)	6 (46.15)	1.71 (0.57–5.18)	0.34	0.56	--	--	
Hispanic White	3 (20.00)	12 (80.00)	0.37 (0.10–1.32)	0.12	0.65	--	--	
Other^a^	22 (44.00)	28 (56.00)	1.08 (0.59–1.98)	0.81	0.31	--	--	
Temperature, n (%)								
<38°C	450 (37.63)	746 (62.37)	REF			--	--	
≥38°C	3 (75.00)	1 (25.00)	5.02 (0.52–48.42)	0.16	1.16	--	--	
Serum WBC^b^, n (%)								
<12,000 cells/μL	404 (37.72)	667 (62.28)	REF			--	--	
≥12,000 cells/μL	47 (43.12)	62 (56.88)	1.29 (0.87–1.93)	0.21	0.20	--	**--**	
SIRS criteria^b^, n (%)								
<2 criteria	411 (37.71)	679 (62.29)	REF			--	--	
≥2 criteria	40 (44.44)	50 (55.56)	1.34 (0.87–2.06)	0.19	0.22	--	--	
E-VW^c^ comorbidity score, median (IQR)	−2 (−7–0)	−3 (−7–0)	1.03 (1.01–1.05)	**<0.01**	0.01	--	--	
Primary psychiatric diagnosis, n (%)								
Depressive disorders	99 (30.75)	223 (69.25)	REF			REF		
Schizophrenia/psychotic disorders	183 (43.26)	240 (56.74)	1.67 (1.22–2.27)	**<0.01**	0.16	1.69 (1.23–2.31)	**<0.01**	0.16
Anxiety and stress-related disorders	27 (32.53)	56 (67.47)	1.10 (0.63–1.93)	0.74	0.29	1.22 (0.69–2.16)	0.45	0.29
Bipolar and related disorders	51 (48.11)	55 (51.89)	2.09 (1.33–3.27)	**<0.01**	0.23	1.94 (1.23–3.07)	**<0.01**	0.23
Personality disorders	14 (19.72)	57 (80.28)	0.56 (0.30–1.06)	0.08	0.32	0.62 (0.32–1.17)	0.14	0.33
Substance use disorders	77 (42.78)	103 (57.22)	1.68 (1.15–2.46)	**<0.01**	0.20	1.83 (1.22–2.74)	**<0.01**	0.21
Other	2 (13.33)	13 (86.67)	0.35 (0.08–1.56)	0.17	0.77	0.38 (0.08–1.78)	0.22	0.79

This table presents the demographic data, clinical variables of interest associated with IAU, and results of both univariable and multivariable logistic regression analyses. Only encounters with a positive UA and without appropriate antibiotic use for a documented urinary indication were included. We only considered antibiotics initiated for urinary indications. Univariable logistic regression estimated using generalized estimating equations was fitted to identify factors associated with IAU, accounting for within-patient correlation across multiple encounters. Variables significant at the 10% level were included in the multivariable model. Forward selection and backward deletion were used to determine the best-fit model using a *P* value of .05 as the inclusion/exclusion criterion. Bolded values denote statistical significance at *P* < .05. ^a^“Other” in the race/ethnicity variable include those who identified as mixed or multiracial, Native American, or Alaskan Native. ^b^Twenty encounters in this cohort lacked serum WBC/SIRS data. ^c^The E-VW score summarizes a patient’s overall comorbidity load, with values ranging from −19 to 89; higher scores indicate greater disease burden.

Abbreviations used: aOR, adjusted odds ratio; E-VW, Elixhauser-van Walraven; IAU, inappropriate antibiotic use; IQR, interquartile range; SE, standard error; SIRS, systemic inflammatory response syndrome; UA, urinalysis; uOR, unadjusted odds ratio; WBC, white blood cell.

## Discussion

Our findings demonstrated that admission UAs led to the diagnoses of either a clinical UTI or ASB in pregnancy in only 1.7% of cases, and most antibiotic courses initiated based on UA results were inappropriate. Although age, sex, and culture positivity are known predictors of antibiotic use^7^ (and were similarly observed in our study), the association with specific psychiatric diagnoses (eg, schizophrenia, manic disorders, and substance use disorders) may reflect contexts where a patient’s verbal output, insight, and reliability may be limited, potentially prompting clinicians to treat bacteriuria out of caution. While it is well established that overuse of UCs drives unnecessary antibiotic use, this study additionally supports emerging work that the urinalysis itself is another key test that can drive inappropriate prescribing.^8,9^ In all, routine ordering of UAs appears to lead to increased inappropriate antibiotic prescribing in patients who are being admitted for psychiatric indications and who do not have clinical evidence of UTI.

While we found no documented cases of CDI in our specified observation period, this aligns with previously published findings and is likely reflective of epidemiologic trends showing a decreasing incidence of hospital-acquired CDI.^7^ When present, increased CDI rates are a convenient surrogate to detect adverse events from antibiotic overuse. However, as it is estimated that patients have a 4% daily risk of adverse drug reactions while on antimicrobial therapy,^10^ it is likely that other, more difficult to measure adverse events went undetected. As such, routinely collecting admission UAs can have a downstream effect that exposes patients to unnecessary risks without clear clinical benefits. Beyond directly affecting patients, indiscriminate UA testing, particularly with reflex to microscopy and culture, drains laboratory personnel time that could be better allocated to more clinically useful tasks.

One limitation of our study is its retrospective nature, which requires exploration of EMR documentation where explicit urinary symptoms may be underreported. Nevertheless, our findings highlight an opportunity for improved stewardship efforts. Education should focus on encouraging judicious use of UAs and reservation of testing only for those with high clinical suspicion of a UTI. Ultimately, our work suggests that routine admission UAs in psychiatric hospitalizations lead to IAU, thus warranting reconsideration of its role in current practice.

## Data Availability

The de-identified data and code supporting the findings of this study are available from the corresponding author upon reasonable request.
